# Lapatinib-loaded human serum albumin nanoparticles for the prevention and treatment of triple-negative breast cancer metastasis to the brain

**DOI:** 10.18632/oncotarget.8697

**Published:** 2016-04-12

**Authors:** Xu Wan, Xiaoyao Zheng, Xiaoyin Pang, Zhiqing Pang, Jingjing Zhao, Zheming Zhang, Tao Jiang, Wei Xu, Qizhi Zhang, Xinguo Jiang

**Affiliations:** ^1^ Department of Pharmaceutics, School of Pharmacy, Fudan University, Key Laboratory of Smart Drug Delivery, Ministry of Education, Shanghai, People's Republic of China; ^2^ Department of Pharmacy, South Campus, Renji Hospital, School of Medicine, Shanghai Jiaotong University, Shanghai, People's Republic of China; ^3^ Shanghai Zhangjiang Medicine Valley Public Service Platform Co., Ltd., Shanghai, People's Republic of China

**Keywords:** brain metastasis, triple-negative breast cancer, lapatinib, human serum albumin nanoparticles, modified Nab technology

## Abstract

Brain metastasis from triple-negative breast cancer (TNBC) has continued to lack effective clinical treatments until present. However, the feature of epidermal growth factor receptor (EGFR) frequently overexpressed in TNBC offers the opportunity to employ lapatinib, a dual-tyrosine kinase inhibitor of human epidermal growth factor receptor-2 (HER2) and EGFR, in the treatment of brain metastasis of TNBC. Unfortunately, the low oral bioavailability of lapatinib and drug efflux by blood-brain barrier have resulted in low drug delivery efficiency into the brain and limited therapeutic effects for patients with brain metastasis in clinical trials. To overcome such disadvantages, we developed lapatinib-loaded human serum albumin (HSA) nanoparticles, named LHNPs, by modified nanoparticle albumin-bound (Nab) technology. LHNPs had a core-shell structure and the new HSA/phosphatidylcholine sheath made LHNPs stable in bloodstream. Compared to free lapatinib, LHNPs could inhibit the adhesion, migration and invasion ability of high brain-metastatic 4T1 cells more effectively *in vitro*. Tissue distribution following intravenous administration revealed that LHNPs (i.v., 10 mg/kg) achieved increased delivery to the metastatic brain at 5.43 and 4.36 times the levels of Tykerb (p.o., 100 mg/kg) and lapatinib solution (LS, i.v., 10 mg/kg), respectively. Compared to the marketed Tykerb group, LHNPs had markedly better inhibition effects on brain micrometastasis and significantly extended the median survival time of 4T1 brain metastatic mice in consequence. The improved anti-tumor efficacy of LHNPs could be partly ascribed to down-regulating metastasis-related proteins. Therefore, these results clearly indicated that LHNPs could become a promising candidate for clinical applications against brain metastasis of TNBC.

## INTRODUCTION

Breast cancer is the most common cancer in women, with more than 1.7 million new cases every year reported by the International Agency for Research on Cancer (IARC) [1]. Although the rapid development of surgical resection, radiotherapy and chemotherapies has successfully controlled the progression of primary breast tumors and prolonged the survival of patients, tumor metastasis has become a major cause of morbidity and mortality in patients with solid tumors. Breast cancer metastasizes primarily to the lung, liver and bones [2]. Brain metastasis represents a late relapse in patients, and the incidence of clinically evident brain metastasis among women with metastatic breast cancer accounts for 25%–34% [3].

Among all breast cancer subtypes, triple-negative breast cancer (TNBC) has the highest incidence of brain metastasis (up to 46%) and the poorest prognosis [4]. The median survival time of TNBC patients with brain metastasis is less than 6 months. Characterized by the absence of the estrogen receptor (ER), the progesterone receptor (PR), and human epidermal growth factor receptor 2 (HER2) [5], both TNBC and its brain metastasis lack specific therapeutic targets. In addition, the blood-brain barrier (BBB) is a formidable obstacle to most potential chemotherapeutic drugs and macromolecule antibodies [6] that are clinically available for brain metastasis of TNBC. Therefore, no standard-of-care therapy has been recommended for patients with brain metastasis from TNBC until present.

In the past few years, many studies have proved that the epidermal growth factor receptor (EGFR) is frequently overexpressed in TNBC [7–9], in association with a high risk of brain metastasis. Accordingly, EGFR could potentially be used in diagnosis and molecular targeting therapy of this breast cancer subtype.

Lapatinib, a selective small-molecule dual-tyrosine kinase inhibitor of HER2 and EGFR, was approved in combination with capecitabine for use in HER2-positive patients with advanced metastatic breast cancer [10]. Its selective EGFR-targeting property suggests that lapatinib might be a candidate drug for brain metastasis from TNBC. Unfortunately, single-agent lapatinib for patients with brain metastasis achieved only 2.6%–6% partial responses [11]. The poor therapeutic effects might be attributed to the low bioavailability of the drug when administered as an oral tablet (Tykerb, unique commercial preparation of lapatinib, GlaxoSmithKline) [12] and to drug efflux by P-glycoprotein (P-gp) and breast cancer resistance protein (BCRP) [13, 14] existing at the BBB, which limit the lapatinib delivery to the brain. Therefore, there is an urgent need for a new therapeutic strategy to enhance the brain delivery of lapatinib and to improve its efficacy against early brain metastasis.

Currently, nanoparticle-based drug delivery has been shown to avoid P-gp-mediated drug efflux, thus elevating drug bioavailability by systemic administration [15, 16]. Moreover, nanoparticles can accumulate at the tumor site because of their enhanced permeability and retention (EPR). Among the existing macro-molecule biomaterials used for the preparation of nanoparticles, human serum albumin (HSA) offers obvious advantages. It is the most abundant plasma protein, with high stability during storage and *in vivo*, and it can be preferentially taken up by tumors [17, 18]. In addition to the EPR effect, HSA nanoparticles can also bind to the 60-kDa glycoprotein (gp60) receptor on vascular endothelial cells, subsequently facilitating the passage of nanoparticles across the endothelial barrier into the tumor. Furthermore, SPARC (secreted protein acidic and rich in cysteine), which is widely present in the extracellular matrix (ECM) of tumor tissue, can attract nanoparticles to inner tumor areas [19, 20], thus resulting in increased anti-tumor activity, such as Abraxane (paclitaxel-loaded HSA nanoparticles) [21]. In addition, lapatinib has a poor water solubility (only 7 μg/mL) and high binding efficacy (> 99%) to albumin. Therefore, HSA nanoparticles could constitute a promising carrier for lapatinib.

Regarding this strategy, we constructed lapatinib–loaded HSA nanoparticles (LHNPs) for treating TNBC brain metastasis. Phosphatidylcholine (PC), a safe material approved by FDA, was introduced into the nano-system to solubilize lapatinib and achieve the appropriate particle size. The possible interaction among the components of the nanoparticles was clarified using differential scanning calorimetry (DSC) analysis and the stability of LHNPs was evaluated in simulated human plasma. Since metastasis is an extremely complicated process involving local invasion, intravasation and extravasation of vascular system, growth at secondary site and angiogenesis [22, 23], adhesion assay, wound healing assay, migration and invasion assay and *in vivo* biodistribution were conducted. Finally, the antitumor efficacy of LHNPs in 4T1 brain metastatic mouse models was investigated, and its mechanisms of action were elucidated by biochemical index assays.

## RESULTS AND DISCUSSION

### Characterization of nanoparticles

**Figure 1:**
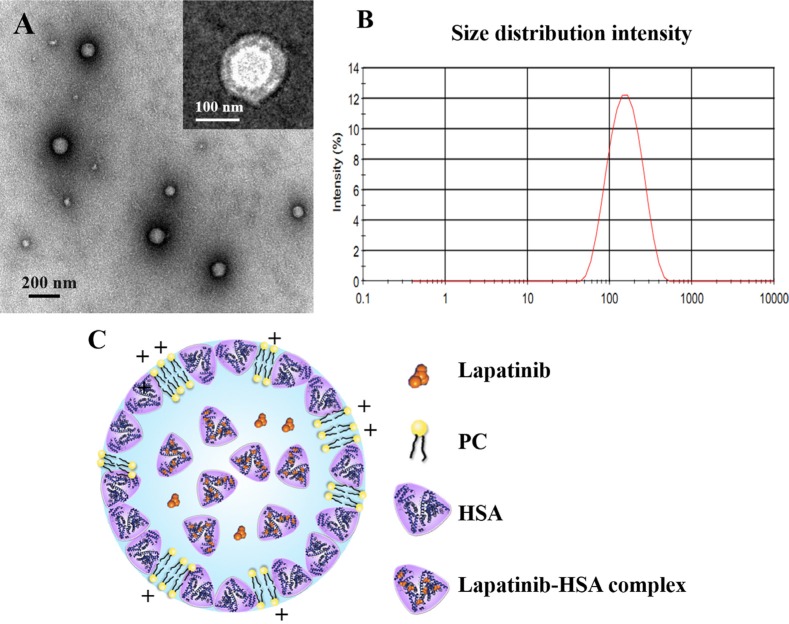
(**A**) Transmission electron micrographs of LHNPs stained with 2% phosphotungstic acid solution or uranyl acetate. (**B**) A representative size distribution profile of LHNPs. (**C**) A schematic figure for LHNPs structure.

Nab technology, a mature technology for nanoparticles industrialization, was modified to extend its range of application and prepare lapatinib-loaded HSA nanoparticles in this study. PC was introduced into the formulation due to the low solubility of lapatinib in chloroform (1.18 mg/mL). The nanoparticles presented spherical and uniform characteristics under transmission electronic microscopy (TEM), with a core-shell structure (Figure 1A). The mean particle size of the LHNPs was approximately 140 nm with a relatively narrow particle distribution (polydispersity index = 0.189, Figure 1B) and the encapsulation efficiency was 84.9 ± 2.45%. LHNPs present positive zeta potential (21.4 ± 0.6 mV), which was primarily due to that the pH of water phase (4~6.5) was below the isoelectric point of PC (pH 6.7).

### DSC analysis

The possible interaction among lapatinib, PC and HSA can be detected by DSC experiments.

**Figure 2:**
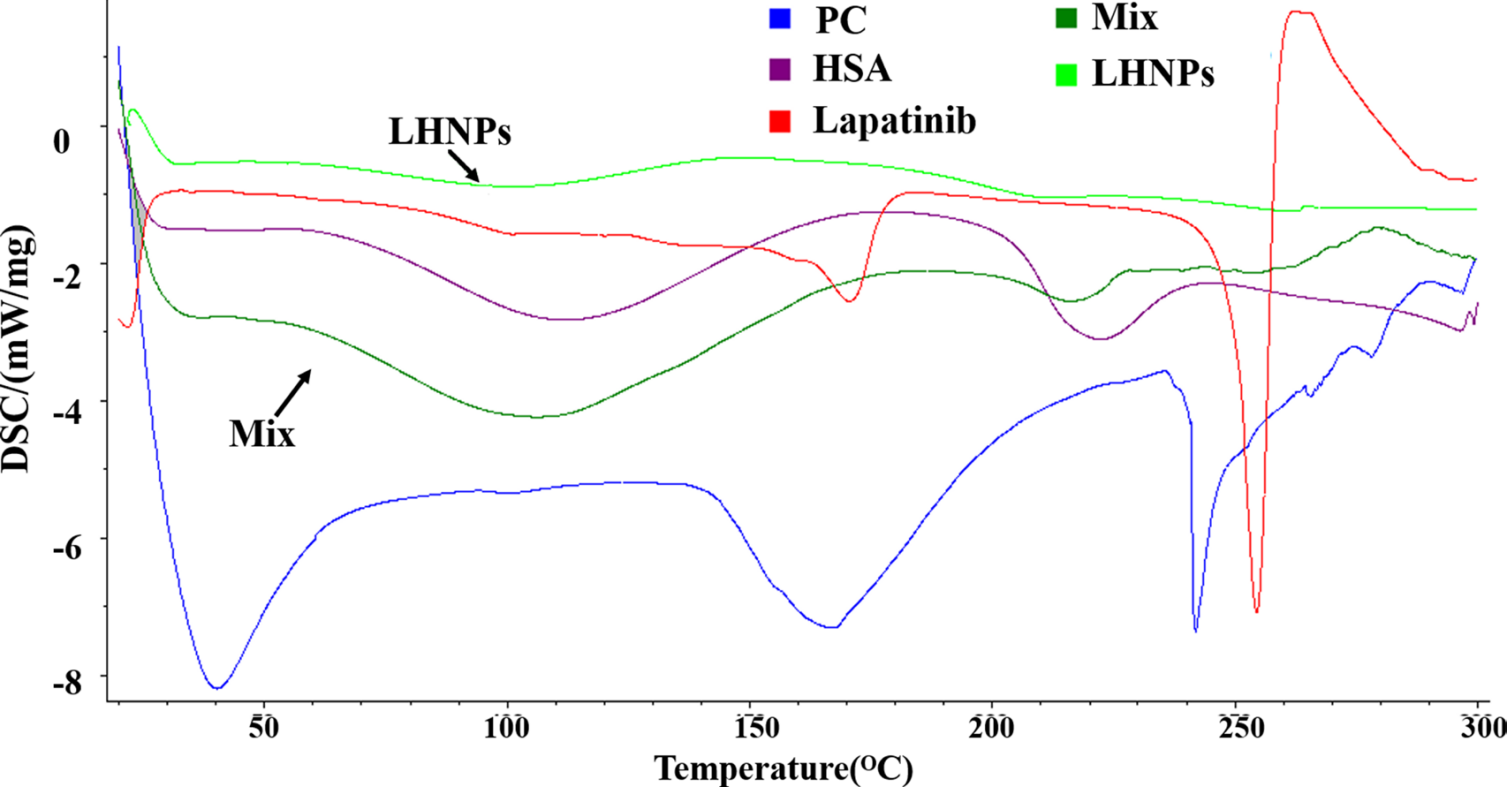
DSC patterns of lapatinib, HSA, PC, physical mixture of three components (Mix) and LHNPs

As shown in Figure 2, the physical mixture of lapatinib, HSA and PC displayed two peaks at approximately 105°C and 215°C, which was quite similar to the HSA. Three endothermic peaks of PC appeared at about 40°C, 168°C and 241°C. No endothermic peaks was observed in DSC thermogram of LHNPs, indicating that lapatinib exist in amorphous state in nanoparticles. Combining the DSC results and transmission electron micrographs, the possible schematic figure for nanoparticles structure was depicted in Figure 1C.

### *In vitro* stability

**Figure 3:**
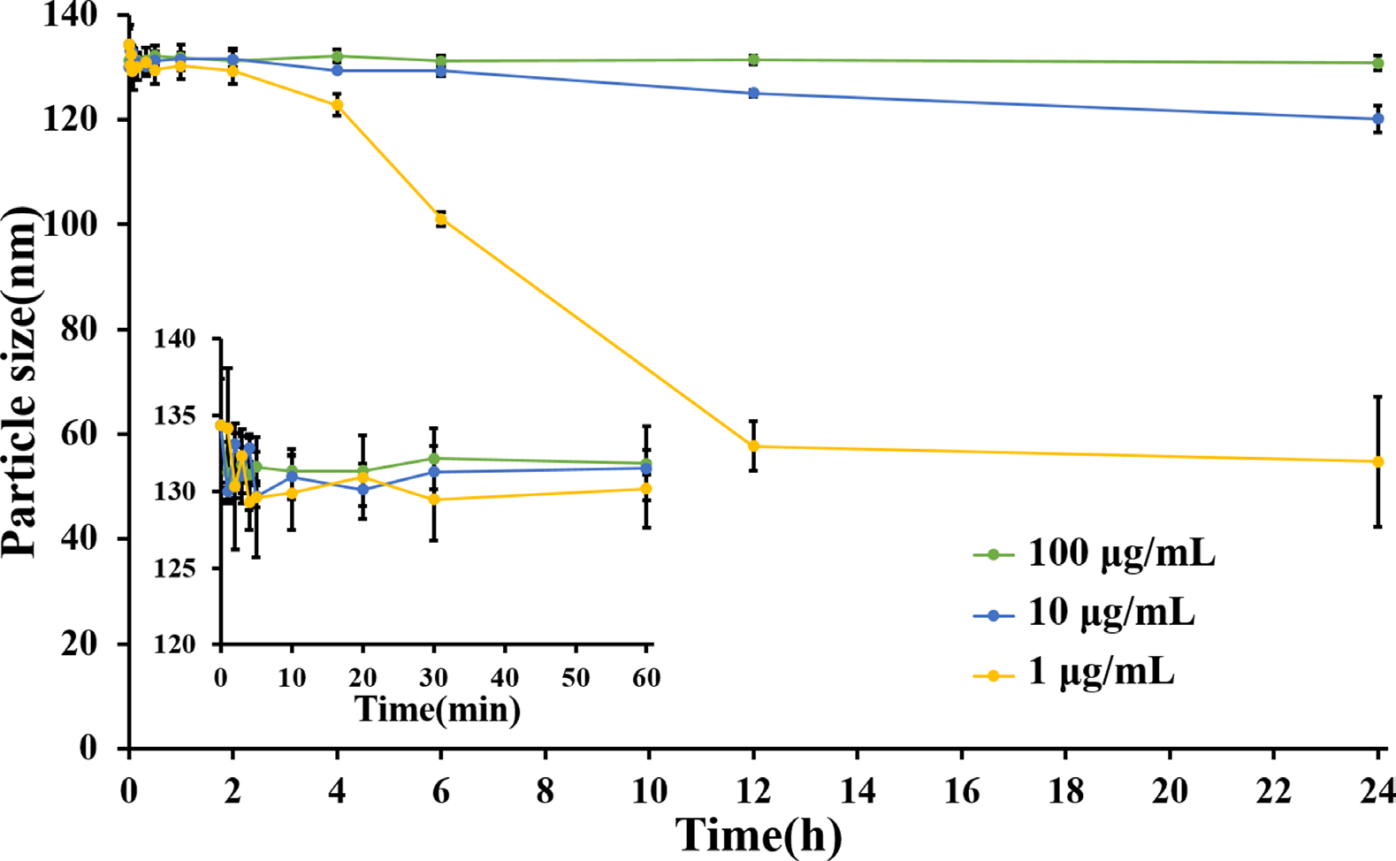
Changes of particle size of different concentrations of LHNPs in 5% HSA medium, which simulated human plasma (*n* = 4)

It was reported that Abraxane was unstable after intravenous injection, and disassociate into individual albumin molecules in several seconds and then circulated in bloodstream as paclitaxel-albumin complexes without desired advantages of nanoparticles [24]. However, LHNPs exhibited strong stability in simulated human plasma at three concentrations similar to the plasma levels of lapatinib at different times after administration (Figure 3). No significant disassociation was observed when the lapatinib concentration was in the range of 10~100 μg/mL during 24 h incubation. When the lapatinib concentration was further decreased to 1 μg/mL, LHNPs still remained stable for 2 h and became a little smaller after 4 h. The significant stability difference between Abraxane and LHNPs could be attributed to the new structure of HSA/PC sheath of LHNPs which enhanced the stability of LHNPs. These results suggested that LHNPs might circulate in bloodstream with integrate nanoparticle structure for hours after intravenous injection, which was essential for LHNPs to exhibit the advantages of EPR effects and protect lapatinib from the BBB efflux.

### Inhibition of metastasis *in vitro*

While the exact mechanism of brain metastasis remains unclear, accumulated studies have proved that the brain metastatic process is a complex process involving multiple steps [25]. First, tumor cells escape from the primary tumor site by losing cell-cell adhesion and gaining motility, enabling them to invade the surrounding tissues [26]. Second, tumor cells degrade the extracellular matrix of the host organ using a variety of proteolytic enzymes [27], and then they move through the matrix and intravasate to enter the systemic circulation. Only a few circulating tumor cells can survive in the systemic circulation and be arrested in the brain by blood flow [28]. Finally, these metastasized cells manage to extravasate through the brain capillaries and embed themselves into the brain parenchyma to form metastasis [29]. To understand the underlying mechanisms and to assess the prevention capacity of LHNPs on brain metastasis, *in vitro* adhesion, migration and invasive assays were performed.

### Inhibition of adhesion ability

**Figure 4:**
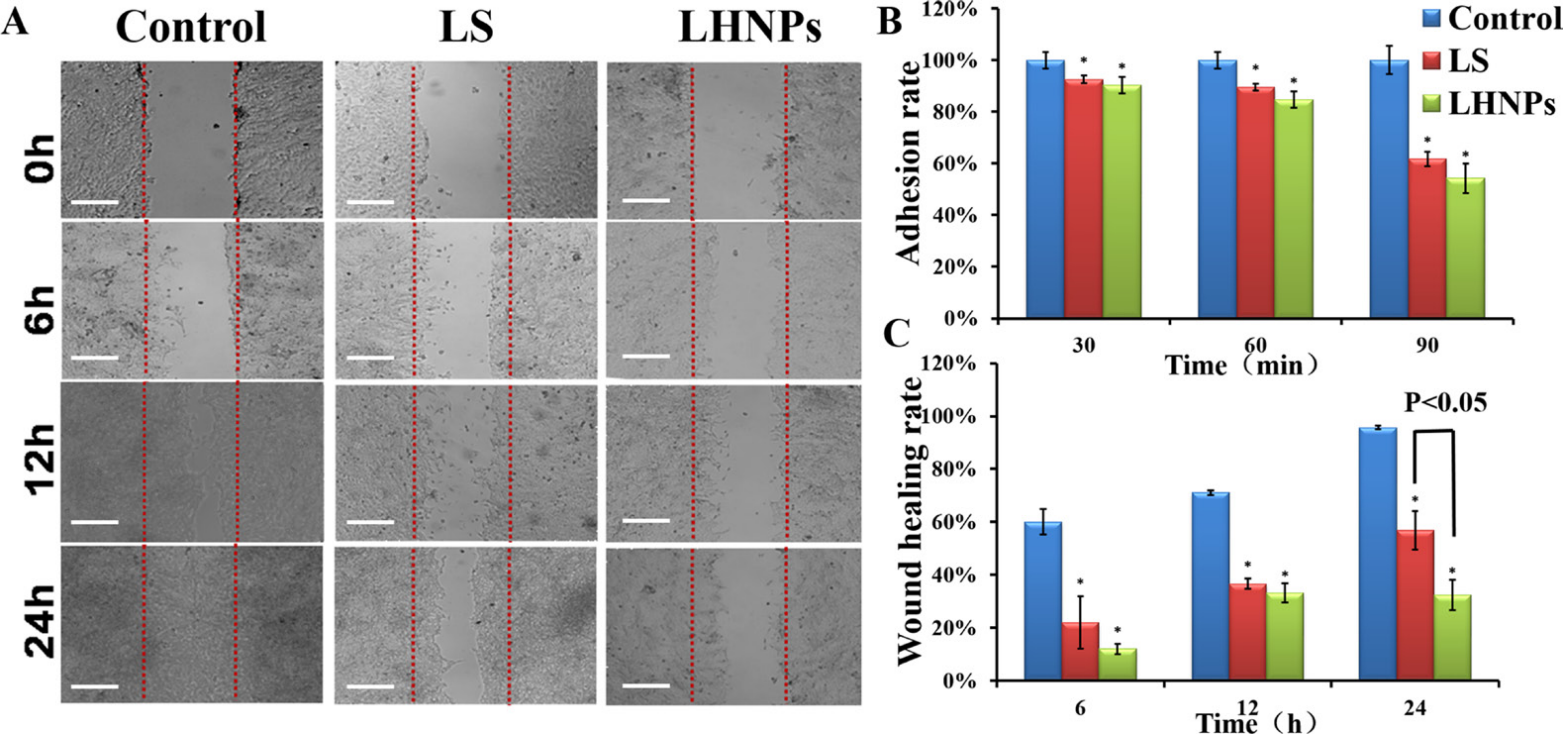
(**A**) Wound healing images after scratch incubation with drug-free medium, LS or LHNPs for 24 h. The bar is 100 μm. (**B, C**) Quantitative analysis of adhesion rate (B) and wound healing rate (C) with different treatments. *p < 0.05, compared with controls.

We examined the influence of LS and LHNPs on the adhesion of 4T1 cells to the plates coated with Matrigel, which is basement member matrix. To avoid interference effects, the concentrations of LS and LHNPs were set at the level of 0.1 μg/mL, which exerted no effects on cell viability as demonstrated in MTT assays [30]. As shown in Figure 4B, the inhibition effects of LS and LHNPs on cell adhesion were exerted in a time-dependent manner, and the LHNPs exhibited slightly stronger inhibitory effects than LS with increasing incubation time (LHNPs, 45.6% vs LS, 38.3% at 90 min).

### Inhibition of migration ability

**Figure 5:**
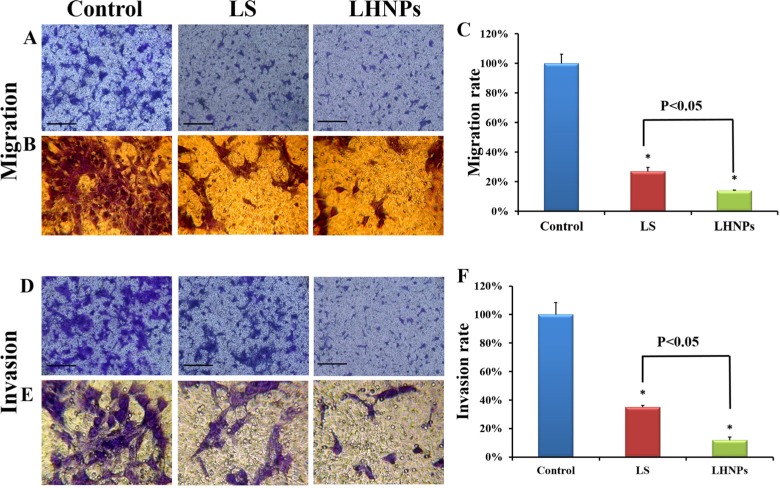
Effects of LS and LHNPs on cell migration and invasion *in vitro* Representative fields of migrated and invasive cells stained by crystal violet after treatment with LS or LHNPs, compared to the control group under a fluorescent microscope (**A, D**) and an optical microscope (**B, E** 400×). Quantitative analysis of the migration (**C**) or invasion rate (**F**) with different treatments. The scale bar represents 100 μm. *p < 0.05, compared with the controls.

In addition to adhesion ability, the motility of tumor cells was also considered an important measurement for evaluating the metastatic potential of cells. Wound healing assay was used to investigate the lateral migration of cells, while transwell assay focused on the vertical mobility. As shown in Figure 4A, the cells in the control group exhibited strong wound healing ability, and they moved rapidly to the blank area of the scratch. After 24 h, the wound healing rate was nearly 100%. In contrast, both LS and LHNPs significantly inhibited cell migration, with healing rates of 55% and 30%, respectively. In transwell assays (Figure 5A, 5B), similar results were obtained. The cellular migration was inhibited to 28.0% by LS and to 18% by LHNPs compared to the control group at 24 h (Figure 5C).

All of these results demonstrated that LHNPs could more effectively inhibit the lateral and vertical mobility of 4T1 cells compared with free drug, which could form the basis for inhibition of the spontaneous metastasis of breast cancer *in vivo*.

### Inhibition of invasion ability

Invasive assay requires tumor cells to migrate through transwell filters precoated with Matrigel, a model of an extracellular matrix barrier. After enzymatically degrading that barrier, the tumor cells traversed the membrane and proliferated in a new location [31]. As shown in Figure 5D and 5E, the cells stained with crystal violet under the filters (invasive cells) were abundant in the view of the control group. However, the invasive cells were visibly decreased with LS and LHNPs treatment. Compared to LS, the inhibition effects of LHNPs were more obvious with the invasion rate decreasing from 38% to 15%, demonstrating that LHNPs displayed significantly higher anti-metastatic ability (Figure 5F).

### *In vivo* model of brain metastasis

**Figure 6:**
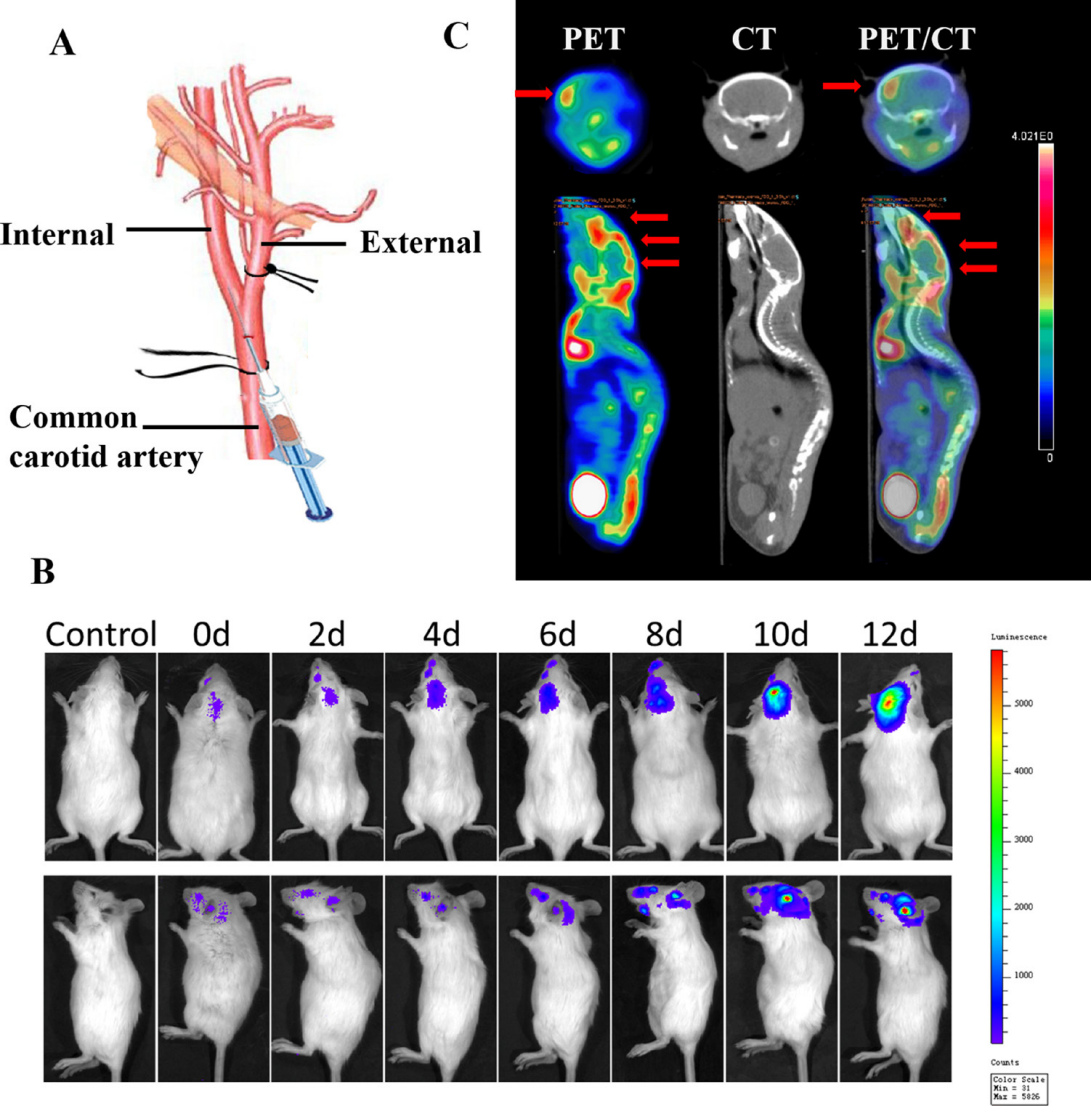
Establishment and confirmation of brain metastasis models (**A**) Illustration of the technique for intracarotid injection of 4T1-luc cells. (**B**) Bioluminescence signal indicating brain colonization by 4T1 cells in a representative mouse at various times after injection of 4T1-luc cells. (**C**) Transverse and coronal sections of PET and CT images of a representative mouse with brain metastasis 4 h after tail-vein injection of ^18^F-FDG. PET/CT fusion image confirms registration of hypermetabolic image on anatomic reference.

Breast cancer cells have been reported to follow a hematogenous route for metastasis to the brain because there is barely a lymphatic system in the brain [32, 33]. Therefore, 4T1-luc cells were injected into the right internal carotid arteries of female BALB/c mice to establish the *in vivo* model of brain metastasis [34, 35]. The method was modified including ligation of external carotid artery, which could greatly prevent the injected tumor cells from colonizing on musculature on the head (supplied by the external carotid artery), and improve the success rate of model construction. As shown in Figure 6B, 4T1 cells with bioluminescence were found to localize in the brain after carotid artery injection and to produce persistent and increasing signals over time, indicating that the majority of the initially arrested cancer cells in the brain survived and displayed aggressively proliferative behaviors. In the PET/CT images in Figure 6C, the hypermetabolic signal in the brain (indicated by red arrows) shows the locations of metastasis and micrometastasis. From the intersecting surface of the brain, a major metastatic lesion with several scattered metastasis was seen in different parts of the brain. These results proved the success of building an *in vivo* model of brain metastasis.

### Tissue distribution *in vivo*

To clarify the tumor-targeting efficacy of LHNPs, the distribution of drugs in normal tissues and in the brain lesions was detected after three formulations of lapatinib treatment. At the same time, to determine the integrity of the BBB in the brain metastasis animal models, the distribution of drugs in normal mice was used as a control. The dose determination of Tykerb and LHNPs was based on the oral dose of human and our pharmacokinetic study, both has similar AUC in plasma (AUC_0–48 h_ 125013.3 ± 4475.3 mg/L × h vs 113718.2 ± 2111.1 mg/L × h) [30], suggesting the doses used to both groups are comparable.

**Figure 7:**
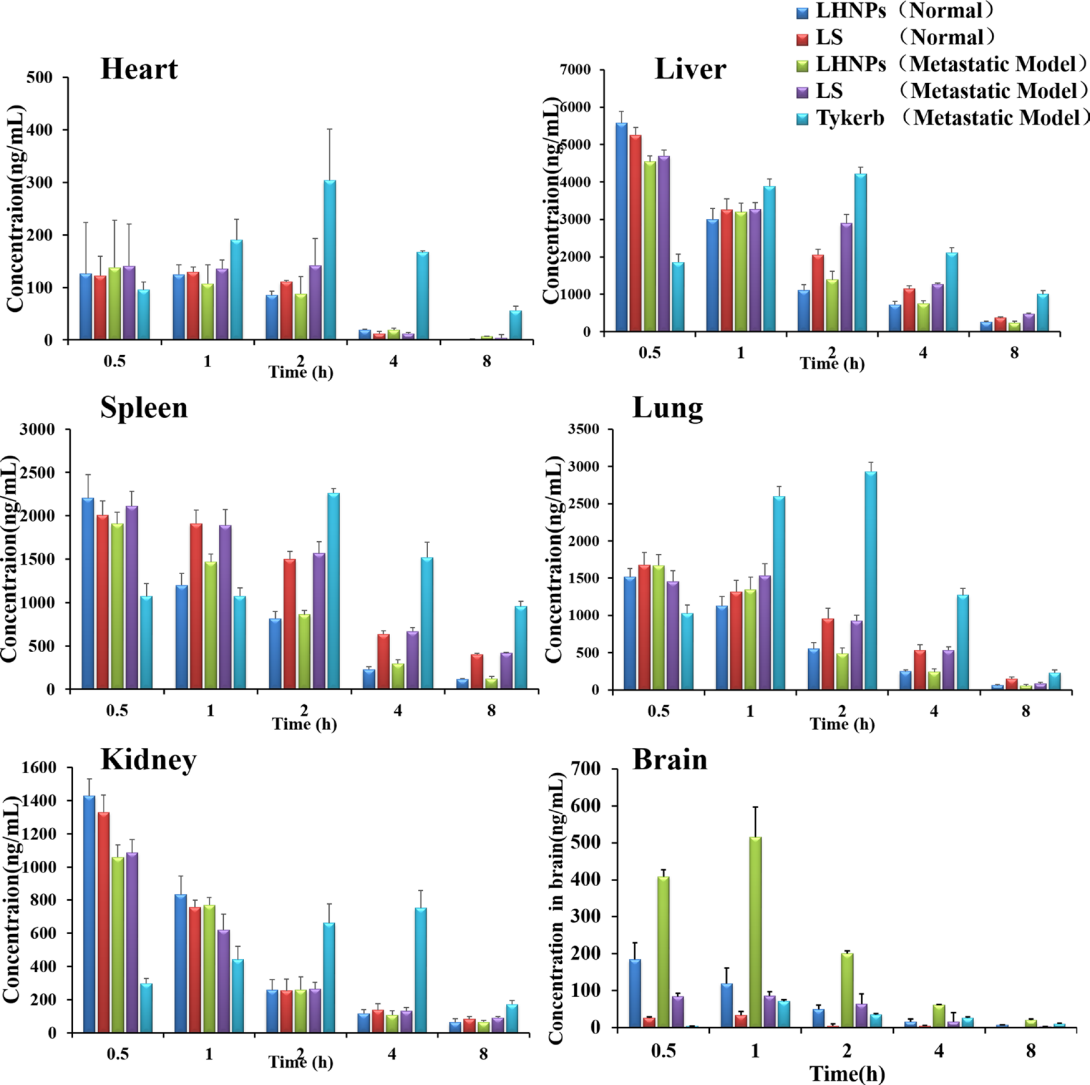
Tissue distribution of lapatinib in mice with brain metastasis at 0.5, 1, 2, 4, and 8 h after administration of Tykerb (p.o. 100 mg/kg), LS (i.v. 10 mg/kg) or LHNPs (i.v. 10 mg/kg) and in normal mice after administration of LS and LHNPs (*n* = 4)

In normal mice, the lapatinib concentrations in the brain in the LS group were quite low and could be negligible after 2 h post-injection (Figure 7), primary due to the drug-efflux effect. Lapatinib has been confirmed to be effluxed by P-gp and BCRP at the BBB [13, 14], thus restricting the entrance of free lapatinib into the brain to exert therapeutic effects. Nevertheless, markedly increased lapatinib concentrations in normal brains were found after LHNPs administration, and the brain AUC of LHNPs was 5.5-fold of that of LS (Table S1), indicating that HSA nanoparticle encapsulation could help to overcome BBB efflux and efficiently deliver lapatinib across the BBB to the brain.

After being administered to the model mice with brain metastasis, LS displayed slightly increased accumulation in the brains, suggesting that the BBB became leaky, along with the progress of brain metastasis. Several researchers have demonstrated that the BBB is intact in and around brain metastases that are smaller than 0.25 mm in diameter, but larger metastasis can affect the integrity of the BBB [36]. This phenomenon was also observed in the LHNPs treatment group, with the drug concentrations in metastatic brains significantly higher than those in normal brains. Notably, lapatinib concentrations of LHNPs in the metastatic brains were the highest at all time points after administration, indicating the stronger penetration ability of LHNPs into the brain regardless of whether the BBB was intact or not. In contrast, Tykerb showed slow initial absorption, resulting in a gradual increase in concentration in all tissues. The lowest concentration was found in metastatic brains, in agreement with the weak therapeutic effects of Tykerb in patients with brain metastasis, as previously reported [11].

The lapatinib accumulations of LHNPs and LS in the brains with metastasis were 5.43 and 1.25 times the level of Tykerb, respectively. In addition, the ratio of AUC_brain_ to AUC_blood_ for LHNPs(0.144) was higher than that for LS(0.050) and Tykerb(0.019) after the effects of plasma concentration was excluded, indicating the great superiority of LHNPs at brain tumor targets *in vivo*, resulting from the advantages of EPR effect and HSA nanoparticles, as stated in the Introduction part.

### *In vivo* anti-tumor and anti-metastasis efficacy

### Survival experiment

**Figure 8:**
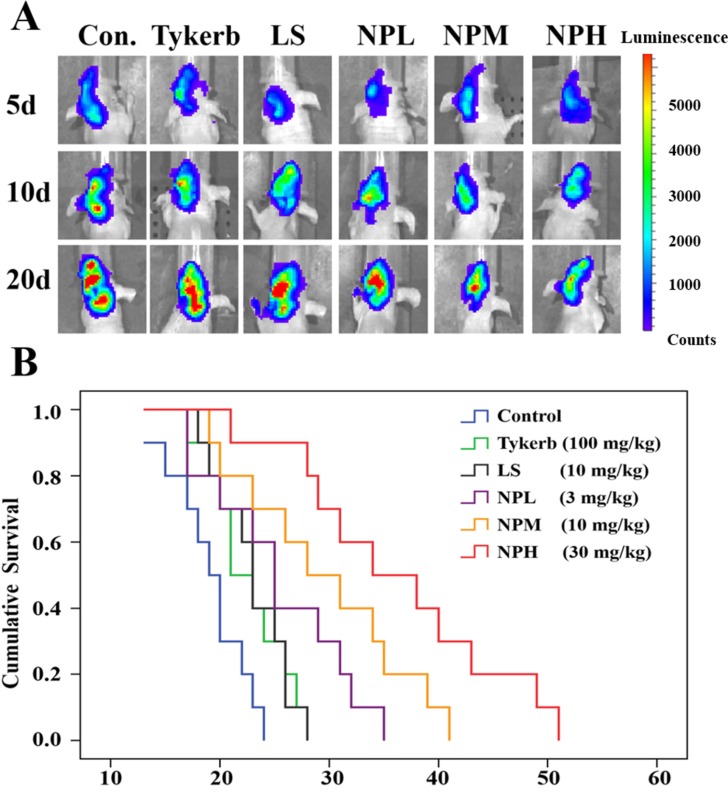
Anti-tumor and anti-metastasis effects of different treatments in 4T1 brain metastatic models (**A**) Bioluminescence signals in metastatic brains of different treatments at 5, 10 and 20 d. (**B**) Kaplan-Meier survival curves of mice with brain metastasis (*n* = 10).

Anti-tumor and anti-metastasis efficacy of LHNPs was evaluated by survival experiment performed on brain metastatic nude mice. As shown in Figure 8A, the Tykerb (100 mg/kg), LS (10 mg/kg) and NPL (3 mg/kg) groups had weak inhibition effects on the bioluminescence signals in metastatic brains, compared with the strongest signals in the control group. However, LHNPs at a medium dose (10 mg/kg) or higher dose (30 mg/kg) not only suppressed tumor growth excellently, but they also efficiently inhibited the further transfer of the established metastasis to other brain regions (data not shown), exhibiting superior therapeutic effects on brain metastasis.

Consistent with the results of bioluminescence analysis, the median survival time of metastatic mice treated with LHNPs at medium and high doses (NPM: 29.6 days; NPH: 36.4 days) was significantly longer than those of mice treated with physiological saline (19.1 days, *p* < 0.05), Tykerb (22.6 days, *p* < 0.05), LS (23.0 days, *p* < 0.05), and NPL (25.4 days, *p* < 0.05) (Figure 8B). Importantly, the total dose of NPH was approximately 10% that used for Tykerb treatment, indicating that the application of LHNPs could reduce the dosage in clinical treatment, with fewer potential side effects compared with Tykerb.

### Histology

**Figure 9:**
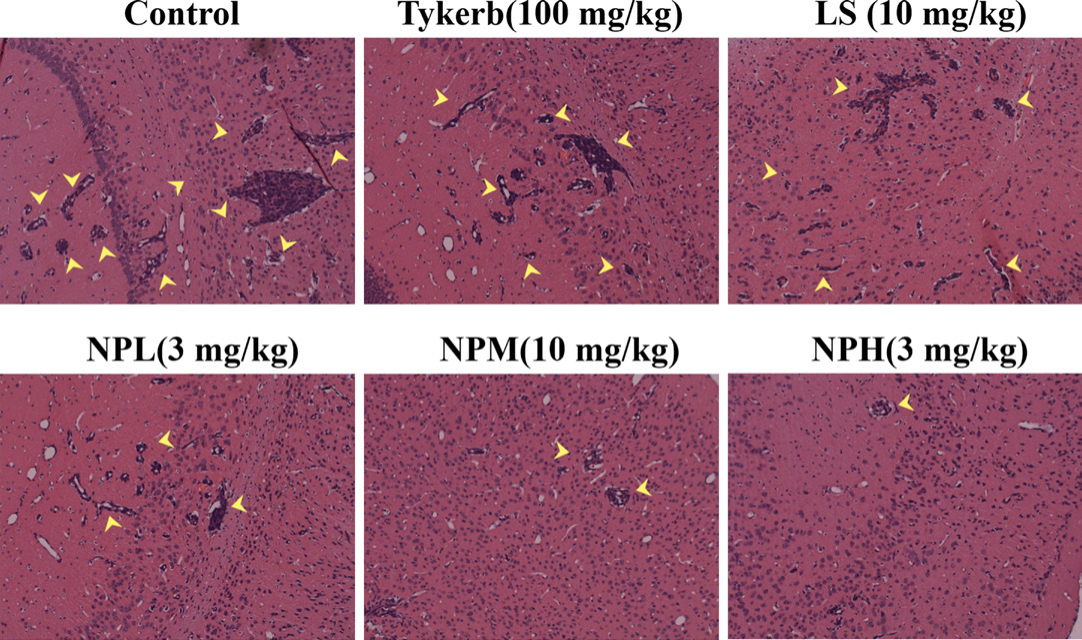
Representative images of H&E staining results of brains after different treatments

At the end of the study, the metastatic brains were excised and imaged. Figure 9 shows that there was abundant brain micrometastasis (marked by yellow arrows) in the control group. Tykerb only slightly decreased the area of brain metastasis, while the number of metastatic lesions and area of total metastatic lesions were visibly decreased with LS or NPL treatment, indicating better inhibition of both injection formulations compared with oral Tykerb. In the groups receiving medium and high doses of LHNPs, fewer brain micrometastases were observed, demonstrating that LHNPs could effectively inhibit tumor metastasis in a dose-dependent manner.

### Measurement of biochemical indexes

Brain metastasis of breast cancer is a complex process involving multiple molecular mechanisms. MMP-2, a classical member of the MMP family, is vital in the progression of intravasation by degrading the extracellular matrix and basement membrane [37]. uPA, a member of the serine protease family, is also involved in the cell-cell adhesion and degradation of the basement membrane by binding to its receptor (uPAR) [38], while OPN expression contributes to 4T1 adhesion activities via mechanisms that are independent of uPA and MMPs [39]. Recently, some chemokines, such as stromal cell derived factor-1α (SDF-1α) and its receptor CXCR4, were suggested to be involved in the progression of specific distant metastasis [40, 41]. In particular, breast cancer cells expressing high levels of CXCR4 could increase their permeability through brain microvascular endothelial cells and facilitate their invasion into the brain [41]. To further explore the molecular mechanisms of LHNPs for brain metastasis, the expression levels of MMP-2, uPA, OPN, and CXCR4 in metastatic brains treated with three formulations were examined by western blotting analysis.

**Figure 10:**
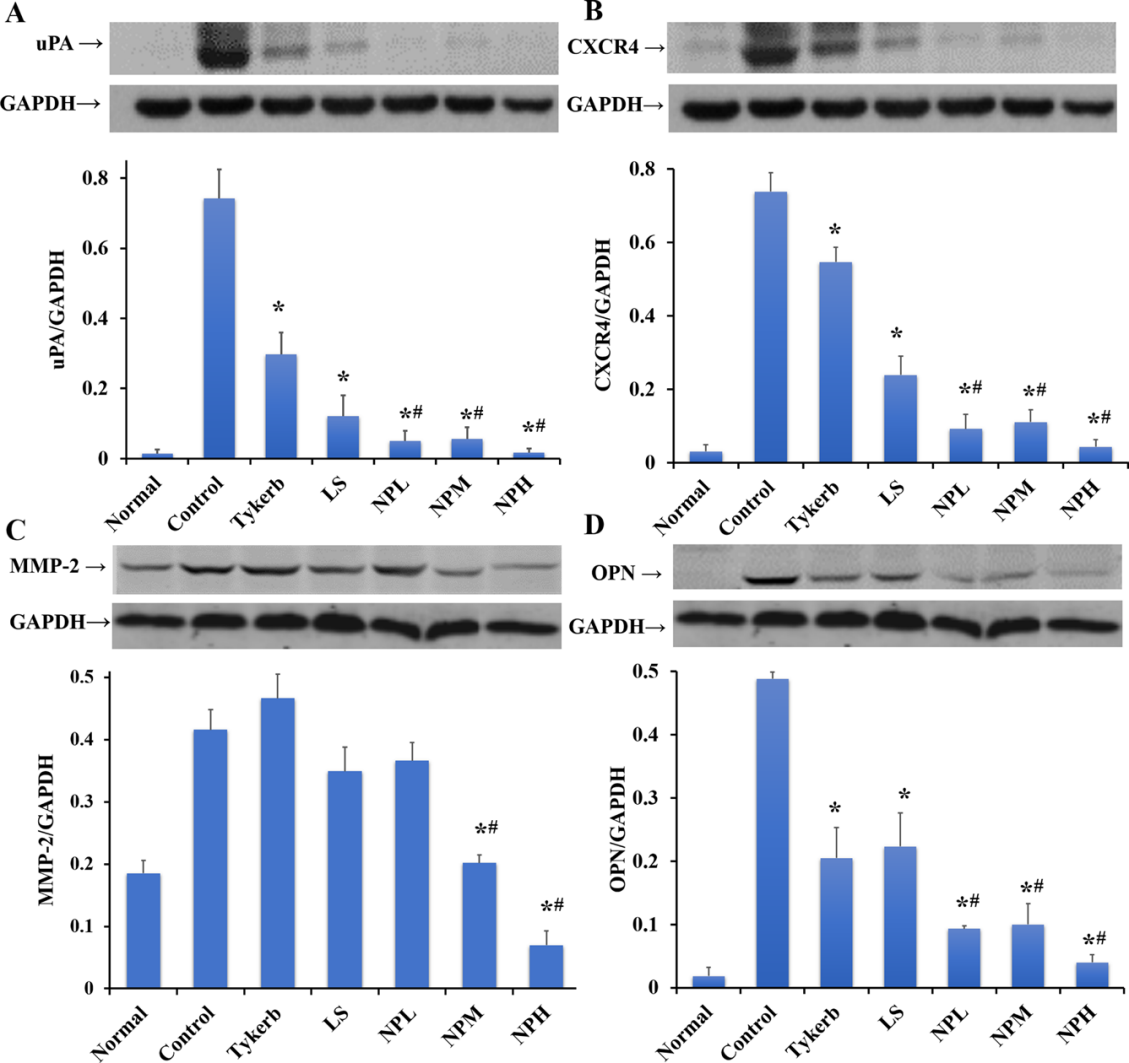
Western blotting of uPA (A), CXCR4 (B), MMP-2 (C) and OPN (D) relative to GAPDH in the brain The data are shown as the means ± S.E.M.s (*n* = 3). *p < 0.05, significantly different from the control group. ^#^*p* < 0.05, significantly different from the Tykerb group.

As shown in Figure 10, overexpression of these biochemical indices was obviously observed in the metastatic brains in the control groups, while the lowest expression appeared in the normal group. The large dosage of Tykerb partly down-regulated uPA, CXCR4 and OPN expression, while LS showed similar or stronger effects in down-regulating the levels of these three proteins, but both formulations had little influence on the overexpression of MMP-2, suggesting that Tykerb and LS might only inhibit the adhesion ability and extravasation of 4T1 cells to some extent. However, significantly better inhibition efficacy was achieved in the groups receiving three dosages of LHNPs, especially NPH group, with the expression levels of the four proteins were at almost the same level as in the normal group, indicating that the incorporation of lapatinib into HSA nanoparticles could enhance drug delivery to brain lesions and further maximize the inhibitive potential of lapatinib itself.

## MATERIALS AND METHODS

### Materials and animals

Lapatinib ditosylate and Gefitinib were purchased from Melonepharma (Dalian, China). Tykerb was purchased from GlaxoSmithKline. Phosphatidylcholine (PC) was purchased from Shanghai Advanced Vehicle Technology Pharmaceutical Ltd. (Shanghai, China). 3-(4,5-dimethyl-2-thiazolyl)-2,5-diphenyltetra-zoplium bromide (MTT), coumarin-6 and Hoechst 33342 dye were purchased from Beyotime (Haimen, China). D-luciferin (potassium salt) was obtained from Gold Biotechnology (St. Louis, USA). The 4T1 cell line was obtained from the Chinese Academy of Sciences Cell Bank. The 4T1-luc cell line was kindly donated by the Shanghai Institute of Materia Medica (Shanghai, China). RMPI medium, fetal bovine serum (FBS) and trypsin-EDTA solutions were obtained from Gibco (CA, USA). All of the other chemicals were analytical or reagent grade.

Female nude Balb/c mice (18–20 g) and ICR mice (20–25 g) were obtained from Shanghai Sino-British Sippr/BK Lab Animal Ltd. (Shanghai, China) and were maintained at a constant temperature (25 ± 1°C). The animal studies were conducted in accordance with the protocols approved by institutional animal care and use committee (IACUC), school of pharmacy, Fudan University.

### Preparation and characterization of nanoparticles

Lapatinib-loaded human serum albumin nanoparticles (LHNPs) were prepared using modified Nab technology. Lapatinib and phosphatidylcholine (PC), at a weight ratio of 1:4, were dissolved in chloroform and then were dripped into 0.6% w/v HSA aqueous solution (pH 4~6.5) under high speed stirring to form an oil-in-water (o/w) emulsion. The primary emulsion was further passed through a Micro fluidizer (Nano DeBEE, USA) 6–14 times at 100–170 MPa. After evaporation by rotary vacuum, the nanoparticle suspension was filtered through filter membranes (Millipore, 220 nm pore diameter) and freeze-dried (Virtis Model Benchtop K, USA).

The particle sizes and zeta potential of the nanoparticles were determined using the Malvern Zetasizer (Malvern, nanoZS, UK), and the morphological examination was performed using transmission electron microscopy (TEOL2010, JEM). The encapsulation efficiency of the LHNPs was measured using High Performance Liquid Chromatography (HPLC) method.

### Differential scanning calorimetry (DSC)

The possible interaction among the components of LHNPs was confirmed by DSC experiments. Approximately 5 mg of the five samples (lapatinib, HSA, PC, LHNPs, and a physic mixture of lapatinib, HSA, and PC) was put in crimped aluminum pans and heated from 20 to 300°C at a rate of 10 K/min under constant purging of nitrogen at 10 mL/min using DSC 204/1/G, Phoenix (Netzsch-Geratebau, GmbH, Selb, Germany).

### *In vitro* stability studies

A variety of ingredients in human bloodstream might induce the dissociation of HSA nanoparticles, thus leading to the reduction of particle size of nanoparticles [42]. Accordingly, the changes of particle size were used to evaluate the stability of LHNPs in bloodstream. An isotonic saline solution containing 5% (w/v) human serum albumin, similar to the main compositions of human plasma, was served as the incubation medium. LHNPs were dispersed in the medium at final concentrations of 1, 10, and 100 μg/mL, respectively, and then incubated at 37°C. The particle size of LHNPs was determined at predetermined time points (*n* = 4) by the Malvern Zetasizer.

### Adhesion assay

In order to better evaluate the efficacy of LHNPs in brain metastasis, 4T1, a highly metastatic murine breast cancer cell line, characterized as ER-/PR-/HER-2 negative and EGFR-expressing, was used to mimic human brain metastasis in this study [43, 44].

The effects of LHNPs on the adhesive ability of 4T1 cells were assayed as reported previously [45, 46] with slight modifications. Briefly, the 96-well plate was coated with Matrigel (BD Biosciences, Bedford, MA, USA) at a concentration of 10 mg/L. Four hours later, adding 1% BSA in PBS for blocking non-specific sites. The 96-well plate was balanced with culture medium for 15 min. Then, untreated 4T1 cells and cells pretreated with LS and LHNPs (lapatinib concentration 0.1 μg/mL) were seeded into precoated plate and were cultured for 90 min. At the indicated time points, unattached cells were removed by washing three times with PBS, and the remaining cells (adherent cells) were numbered by colorimetric MTT assay.

### Migration assays

### Wound-healing assay

The effects of LHNPs on cell lateral migration ability were assessed by wound-healing assay [47]. 4T1 cells were seeded into a 6-well plate and were cultured to 90% confluence in complete medium. The wounded lines were created by applying a plastic pipette tip (1 mm) to the cell monolayer across the center of each well. After rinsing with PBS three times to remove cell debris, the wounded monolayers were incubated in the cell culture medium with or without LS or LHNPs (the lapatinib concentration was 0.1 μg/mL) for 24 h. Images of cell migration into the wound surface were obtained under an inverted microscope at the indicated time points, and the wound healing rate was calculated as the ratio of wound width in the experimental group to that in the control group, using Image J software.

### Transwell migration assay

Transwell migration assay was used to test the vertical movement of cells. A total of 5 × 10^5^ cells in 100 μL were plated in the top chamber of 24-well transwell (8-μm pore size, Corning, N.Y., USA). Before seeding, cells were plated in serum-free culture medium supplemented with 0.1% BSA and pre-incubated with LS or LHNPs (lapatinib concentration of 0.1 μg/mL) for 24 h, respectively. The lower chamber contained culture medium with 10% FBS, which served as a chemoattractant. After 24 h of incubation at 37°C, the migrated cells remaining on the lower surface of the insert membranes were fixed and stained with crystal violet solution for 30 min at room temperature. After drying in the air, the migrated cells were photographed in five random fields, and quantified by absorbance at 570 nm in a plate reader after washing with 33% acetic acid, respectively.

### Invasion assay

For cell invasion, the upper surfaces of transwell filters were precoated with Matrigel (60 μL) as protocols for adhesion assay. Then, pretreated 4T1 cells (2×10^6^/mL) with LS or LHNPs (lapatinib concentration 0.1 μg/mL) were suspended in 100 μL of serum-free culture medium and were added to the tops of the Matrigel-coated transwell filters. Cells incubated without any treatment served as the controls. All of the cells were incubated at 37°C and were allowed to invade for 24 h. After incubation, cells invaded through the transwell filters were fixed, stained and counted as per the protocols for migration assay.

### *In vivo* model of brain metastasis

For *in vivo* imaging, 4T1 cells were stably transfected with firefly luciferase (Luc) in a lentiviral construct. Luc-tagged cancer cells (4T1-luc) were injected into the right internal carotid artery of female BALB/c mice, as per a protocol described previously [30, 31] with slight modifications. Briefly, the mice were anesthetized with chloral hydrate, and then the right common carotid artery of the mice was carefully exposed, separated from the vagal nerve and tied loosely with a surgical ligature (Figure 6A). The external carotid artery branch was also separated and was tied tightly with another surgical ligature. Then, 4T1-luc cells (2 × 10^7^/mL) were suspended in PBS at a total volume of 100 μL and were slowly injected into the internal carotid artery from the common carotid artery using a small syringe with a 32 G needle (Becton Dickinson, Franklin Lakes, NJ). Subsequently, the ligature of the common carotid artery was pulled for hemostasis and was then removed before the wound was sealed.

The location and growth of brain metastasis were monitored by repeated noninvasive bioluminescence imaging, using an IVIS Spectrum Imaging System (Caliper) after luciferin injection (i.p. 0.15 g/kg,) on days 0, 2, 4, 6, 8, 10 and 12 after cell injection. To confirm the successful establishment of brain metastasis models, *in vivo* PET/CT was used for metastasis detection (Supplementary Information).

### Biodistribution studies

To clarify the tumor-targeting efficacy of LHNPs, the concentration of lapatinib in different tissues was measured after administration. Sixty brain metastasis mice were randomly divided into three groups, receiving Tykerb (p.o., 100 mg/kg), LS (i.v., 10 mg/kg) or LHNPs (i.v., 10 mg/kg). To determine the integrity of the BBB in brain metastasis animal models and the penetration ability of LHNPs across the BBB *in vivo*, 30 normal mice were also divided into two groups, receiving LS or LHNPs at same dose, and they served as control groups. Subsequently, the mice were sacrificed, and blood and tissues (heart, liver, spleen, lungs, kidneys and brain) were collected at 0.5, 1, 2, 4, and 8 h after drug administration.

Lapatinib concentrations in plasma and tissue samples were analyzed by LC/MS/MS [32]. Then the area under the blood or tissue concentration versus time curve (AUC_0-t_) was calculated using the trapezoidal method.

### Effects of the lapatinib formulations on brain metastasis model mice

### *In vivo* inhibition of brain metastasis

Nude brain metastasis mice were established as described above. After being examined under the IVIS Spectrum Imaging System (Caliper), mice with similar bioluminescence signals were randomly divided into six groups (*n* = 16). The mice were injected with NPH (LHNPs, 30 mg/kg), NPM (LHNPs, 10 mg/kg), NPL (LHNPs, 3 mg/kg), LS (10 mg/kg) or saline (control group) via the tail vein at 3-day intervals. The compared group was orally administered Tykerb (100 mg/kg) every day. Bioluminescence signals were recorded at days 5, 10, 20, and the inhibition efficacy of brain metastasis for each formulation was evaluated by measuring the survival time of the animals after the treatments.

### Histology

On 20th day after treatment, three mice randomly selected from each group were sacrificed and fixed by heart perfusion with 4% paraformaldehyde solution, and their brains were harvested, fixed in 4% paraformaldehyde, embedded in paraffin, and sectioned. The sections were stained with hematoxylin and eosin (H&E) and were observed using an optical microscope.

### Measurement of biochemical indexes

Animals (*n* = 3 for each group) were anesthetized and transcardially perfused with saline. The samples of brain metastasis were rapidly isolated and stored at −80°C. The expressions of urokinase-type plasminogen activator (uPA), osteopontin (OPN), matrix metalloproteinase-2 (MMP-2) and CXCR4 in the location with brain metastasis were determined using western blot analysis. The same location in the brains of normal mice was also analyzed as a negative group. The following antibodies were used: anti-urokinase-type plasminogen activator (uPA) (Santa Cruz Biotechnology, USA), anti-OPN (Abcam, USA), anti-matrix metalloproteinase-2 (MMP-2, Epitomics, USA), and anti-CXCR4 (Abcam, USA).

### Statistical analysis

All of the data are expressed as the mean ± S.D. One-way ANOVA, followed by Dunnett's *post hoc* analysis, was used for multi-group comparison. Student's *t*-test was used for two-group comparison. Statistical significance was set at *p* < 0.05.

## CONCLUSIONS

In the present study, we prepared lapatinib-loaded HSA nanoparticles which have much possibility for clinical applications due to the safe adjuvants and mature Nab-technology for industrialization. The *in vitro* experimental results demonstrated that LHNPs could remain the integrated structure of nanoparticles in bloodstream for hours and effectively inhibit the adhesion, migration and invasion ability of 4T1 cells. Following intravenous administration in 4T1 brain metastatic mice, LHNPs could remarkably increase lapatinib accumulation in metastatic brains and down-regulate metastasis-related proteins, which could be beneficial for blocking the metastasis process and reducing the occurrence of underlying distant metastasis. Additionally, increased drug delivery to the brain maximized the cytotoxic potential of lapatinib to cancer cells and exhibited excellent therapeutic effects *in vivo*. All of these results suggested that LHNPs might be a promising candidate for the prevention and treatment of brain metastasis of TNBC.

## SUPPLEMENTARY MATERIALS TABLES


