# Supplementary material for: Lapatinib-loaded human serum albumin nanoparticles for the prevention and treatment of triple-negative breast cancer metastasis to the brain
Source: Oncotarget. 2016 Apr 12;7(23):34038–51. doi: 10.18632/oncotarget.8697 (PMC5085136; doi:10.18632/oncotarget.8697)
Supplement: Supplementary file 1 [file oncotarget-07-34038-s001.pdf]

Lapatinib-loaded human serum albumin nanoparticles for the prevention and treatment of triple-negative breast cancer metastasis to the brain

Supplementary Materials

SUPPLEMENTARY 1

On the 12th day post-cell injection, the mouse models were injected with 7.4-MBq of ^{18}F-FDG via the tail vein, and clearance of nonspecifically bound tracer was allowed for 4 h. After anesthesia with isoflurane, the mice were then scanned with a small animal-dedicated micro-PET/CT system (Inveon small-animal PET/SPECT/CT system, Siemens Medical Solutions, USA, Inc.). The acquired PET images were merged with the CT images to ensure the actual location of brain metastasis, where the FDG were highly unabsorbed.

SUPPLEMENTARY 2

Table 1: The area under the blood or tissue concentration of lapatinib versus time curve ($AUC_{0-8\ h}$)

Tissues	LHNPs (normal) (iv, 10 mg/kg)	LS (normal) (iv, 10 mg/kg)	LHNPs (model) (iv, 10 mg/kg)	LS (model) (iv, 10 mg/kg)	Tykerb (model) (po, 100 mg/kg)
Heart	342.7 ± 23.56	362.61 ± 9.45	349.40 ± 32.33 [b, c]	427.47 ± 39.35	1257.93 ± 67.15
Liver	9373.24 ± 161.27[a]	12330.05 ± 147.80	9482.81 ± 179.23 [b, c]	13870.97 ± 181.32	18509.93 ± 141.83
Spleen	4131.23 ± 95.04[a]	7392.05 ± 88.95	4466.11 ± 53.81[b, c]	7647.30 ± 117.43	11207.13 ± 56.22
Lung	3290.83 ± 75.35[a]	5159.73 ± 110.78	3413.41 ± 81.53 [b, c]	5030.11 ± 100.81	11133.77 ± 95.87
Kidney	2195.49 ± 61.71	2188.10 ± 53.97	1939.33 ± 57.16[c]	1970.12 ± 45.60	4065.58 ± 83.43
Brain	316.18 ± 24.65[a]	57.46 ± 6.85	1113.04 ± 28.36[b, c]	255.11 ± 18.64	204.79 ± 13.30
Blood	7407.73 ± 142.54[a]	5002.11 ± 115.61	7712.01 ± 165.79 [b, c]	5054.23 ± 170.98	10714.35 ± 123.54

[a]$p < 0.05$, compared with LS (normal). [b]$p < 0.05$, compared with LS (model). [c]$p < 0.05$, compared with Tykerb.
